# Mono- and bi-functional arenethiols as surfactants for gold nanoparticles: synthesis and characterization

**DOI:** 10.1186/1556-276X-6-103

**Published:** 2011-01-27

**Authors:** Floriana Vitale, Ilaria Fratoddi, Chiara Battocchio, Emanuela Piscopiello, Leander Tapfer, Maria Vittoria Russo, Giovanni Polzonetti, Cinzia Giannini

**Affiliations:** 1 ENEA (Italian National Agency for New Technologies, Energy and the Sustainable Economic Development), UTTMATB (Technical Unit of Materials Technologies - Brindisi), Brindisi Research Centre, S.S. 7 Appia km. 706, 72100 Brindisi, Italy; 2 Dept. of Chemistry, University of Rome "La Sapienza", P.le A. Moro n. 5, 00185 Roma, Italy; 3 Dept. of Physics, INFM, INSTM and CISDiC Unit, University "Roma Tre", Via della Vasca Navale n. 84, 00146 Roma, Italy; 4 Istituto di Cristallografia (IC), CNR, via Amendola 122/O, 70126 Bari, Italy

## Abstract

Stable gold nanoparticles stabilized by different mono and bi-functional arenethiols, namely, benzylthiol and 1,4-benzenedimethanethiol, have been prepared by using a modified Brust's two-phase synthesis. The size, shape, and crystalline structure of the gold nanoparticles have been determined by high-resolution electron microscopy and full-pattern X-ray powder diffraction analyses. Nanocrystals diameters have been tuned in the range 2 ÷ 9 nm by a proper variation of Au/S molar ratio. The chemical composition of gold nanoparticles and their interaction with thiols have been investigated by X-ray photoelectron spectroscopy. In particular, the formation of networks has been observed with interconnected gold nanoparticles containing 1,4-benzenedimethanethiol as ligand.

## Introduction

The potential application of small metal particles as functional units in innovative optoelectronic devices has inspired recent research on their synthesis and properties [1]. Very stable metal nanoparticles have been devoid of sufficient chemical reactivity to allow the preparation of interconnected one- or two-dimensional structures forming quantum wires and quantum wells [2]. A variety of techniques have been proposed for the preparation of colloidal solutions of metallic nanoparticles, ranging in size from 1 to 100 nm. Among others, it is noteworthy the use of reverse micelles as reaction vessels in which the size of the nanoparticles is determined by the water content of the vesicle [3]. The classic citrate method for the generation gold colloids [4] has been recently exploited for the preparation of gold nanowires [5]. However, these techniques suffer serious drawbacks; in fact depending on the preparative conditions, the particles have a tendency to agglomerate slowly and lose their dispersed character.

The pioneering Brust's synthesis [6] of thiol-derivatized gold nanoparticles in a *two-phase *liquid-liquid system allowed obtaining stable self-assembled monolayers. Thiol surfactant imparted stability to nanoparticles without coagulation or loss of solubility, even after several months of storage in air at room temperature. The size, shape, surface functionality, and solubility of the nanoparticles could also be tailored. Since the formation of gold nanoparticles is thermodynamically favorable, the gold/solution interfacial energy is lowered by the chemisorption of a thiol monolayer onto the surface of the gold nanoparticles [7,8].

A method for the preparation and isolation of all-aromatic monolayer-protected gold-nanoparticles thiolates (MPCs) has been recently disclosed [9]. Thiolate-passivated MPCs are nanometer-scale, metal-core entities tending toward molecular precision. Their exceptional electronic and chemical properties have attracted great interest in both fundamental and applied chemical research. All-aromatic MPC variants are of potential value for enhanced rates of electron and excitation transfer, and benzenethiolates (-SC_6_H_5_) are prototypical for this class of materials. Complete conjugation gives a maximally coupled electronic structure. These substances are very promising as crystalline materials and interesting to further improve our knowledge on the gold-thiolate bonding interaction [10]. Materials in size and composition similar to gold-arenethiolate compounds have been reported by Murray et al. [11]; this author has recently published a review that highlights the main features concerning gold nanoparticles, with emphasis on the optical, electrochemical, and catalytic properties [12]. Within this topic, advances in the synthesis methods allowed to attain the control of shape and dispersion with a significant improvement of catalytic activity [13]; in particular ligand-stabilized Au_55 _clusters that constitute an array of quantum dots [14] and truly monodisperse nanoparticles with precise number of Au atoms (144) and thiolate ligands (60) have been achieved through a two-step method featuring a thiol etching of the preformed nanoparticles [15]. The synthesis approach is mainly devoted to achieve Au nanoparticles with dimensions ranging from 1.5 to a few of nanometers. However, for biomedical applications, there is the need of thiol protected AuNPs with core diameters greater than 5.0 nm; this goal has been reached by the use of Bunte salts as functional ligands [16].

Much of recent research has involved the characterization of collective properties of disordered and crystalline two-dimensional (2D) and three-dimensional (3D) arrangements of clusters [17]. In the literature, two synthetic strategies in making nanoscale gold assemblies or networks are reported: *in situ *formation of nanoscale gold aggregates by chemical linkages between molecules and self-aggregation among already formed gold particles. For example, Au sponges of finely branched self-assembled nanowires, whose diameters can be selected in the range 15-150 nm, have been prepared [18]. The solution-phase assembly of gold particles into relatively linear chains of fairly controllable length of up to 1 μm has been achieved by molecularly linking nanoparticles with alkanedithiols. The growth process can be controlled to prepare dimers, oligomers, and polymer-like gold nanoparticle chains by varying the ratio of alkanedithiols to nanoparticles [19]. The surface plasmon coupling of regularly spaced gold could be of interest in the fabrication of optical waveguide and nanoelectronic systems.

The functionalization of the gold nanoparticles with an aromatic thiol, i.e., benzylthiol, is inserted into this framework and motivated from the need of giving more structural rigidity and compactness to the thiol-gold nanoparticle systems, being this ligand about 1.25 nm long only. The use of a bi-functional thiol would in principle allow having a double linkage with two different gold nanoparticles. The aim of our research has been to achieve gold nanoparticles stabilized by different mono- and bi-functional arenethiols, namely, benzylthiol (BzT) and 1,4-benzenedimethanethiol (BDMT).

In this article, the synthesis, and microstructural and morphological characterizations of arenethiol-functionalized gold nanoparticles are reported. The investigations have been performed by means of high-resolution transmission electron microscopy (HREM), X-ray powder diffraction XRD (XRPD), and the chemical bonding and surface functionalization have been assessed by X-ray photoelectron spectroscopy (XPS).

## Experimental section

### Materials and chemicals

The functionalized gold nanoparticles have been synthesized at room temperature (RT). Deionized water has been obtained from Millipore Milli-Q water purification system. Hydrogen tetrachloroaurate (III) trihydrate (Aldrich, 99.9+%), benzylthiol, BzT (Aldrich, 99%), 1,4-benzenedimethanethiol, BDMT (Aldrich, 98%), tetraoctylammonium bromide, TOAB (Aldrich, 98%), sodium borohydride (Aldrich, 99%), Li(C_2_H_5_)_3_BH (superhydride) (Aldrich, 99%), sodium sulfate anhydrous (Carlo Erba), celite 545 filter agent (Aldrich), and the other organic solvent (Aldrich reagent grade) have been used as received.

### Synthesis of gold nanoparticles

(i) Gold nanoparticles stabilized with benzylthiol have been synthesized by means of the modified Brust's two-phase procedure [20], with Au/S ratios 0.75:1 (sample **A1**), 1.5:1 (sample **A2**), 6:1 (sample **A3**), 15:1 (sample **A4**).

**Figure 1:**
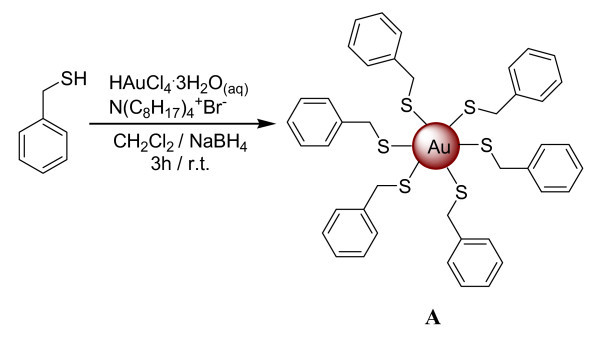
**Scheme 1**. Reaction scheme of the modified two-phase synthesis, based on the Brust's procedure, to obtain benzylthiol-capped gold nanoparticles (synthesis i, sample **A**).

**Figure 2:**
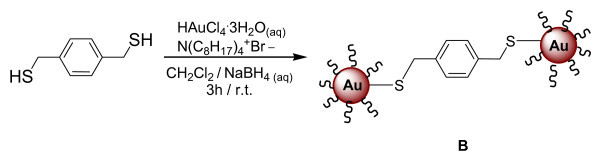
**Scheme 2**. Synthetic scheme of 1,4-benzenedimethanethiol-capped gold nanoparticles (sample **B**).

The reaction route leading to the benzylthiol-capped gold nanoparticles is shown in Scheme 1 (Figure 1, sample **A**). Using the Au/S ratio 0.75:1 as an example: HAuCl_4_·3H_2_O (0.350 g, 0.88 mmol) was dissolved in 30 ml of H_2_O and added to a solution of BzT (0.145 g, 1.17 mmol) in 80 ml of CH_2_Cl_2_, dichloromethane. The gold ions were transferred from the aqueous phase into CH_2_Cl_2 _upon addition of 1.6 g of tetraoctylammonium bromide (TOAB), and the mixture was vigorously stirred at RT. The gold was reduced upon addition of 22.3 ml of a 0.4 M aqueous solution of NaBH_4, _added over a period of 1 min to the vigorously stirring solution. The solution was left stirring for a further 3 h. Then, 50 ml of H_2_O and 50 ml of CH_2_Cl_2 _were added, and the organic phase was separated and dried over anhydrous sodium sulfate. The drying agent and any insoluble materials were removed by filtration and the solution was reduced to a solid, in vacuum. The dark brown residue was resuspended in methanol with the aid of sonication and stirred for 2 h. The suspension was filtered over Celite and subsequently washed with 400 ml of methanol and 400 ml of acetonitrile to remove any excess of arenethiolate, TOAB, and by-products. The product was obtained by washing off the Celite with CH_2_Cl_2_, then solvent was removed in vacuum, and the pure product was dried overnight. The yield for sample **A1 **was 87% (0.3045 g of obtained product).

(ii) The one-phase synthesis of benzylthiol-capped gold nanoparticles was also performed as a comparison, following a published procedure [21], using the following Au/S ratios: 1.07:1 (sample **A5**), 1.07:1 (sample **A6**), 2.15:1 (sample **A7**), 10.7:1 (sample **A8**). The reaction with 1.07:1 ratio is described in detail. A colorless solution of benzylthiol, BzT, (20 μl), and a bright yellow solution of HAuCl_4_·3H_2_O (0.09 M, 1.2 ml) were mixed in THF. After stirring 2 h at room temperature, the color of solution changed to opaque orange. Then, 1.15 ml 1.0 M solution of Li(C_2_H_5_)_3_BH (superhydride) in THF was added drop by drop over a period of 2 min with gas evolution and a color variation of the solution to dark red-brown was observed. The mixture was evaporated to 1 ml and transferred into a centrifuge tube, yielding BzT-AuNPs. For the purification, cold absolute ethanol (40 ml) was added to the BzT-AuNPs; this suspension was kept at *T *= -18°C overnight and then centrifuged to get the dark brown precipitate, thoroughly washed with ethanol and dried in vacuum.

(iii) The synthesis of gold nanoparticles passivated by BDMT was performed using the Au/S ratio 0.75:1, sample **B**. HAuCl_4_·3H_2_O (tetrachloroauric acid, 0.175 g, 0.44 mmol) was dissolved in 15 ml of H_2_O and added to a solution of BDMT (0.999 g, 0.58 mmol) in 40 ml of CH_2_Cl_2_. The aqueous phase was transferred into CH_2_Cl_2 _upon addition of 0.800 g of TOAB, and the mixture was subjected to vigorous stirring. The gold was reduced upon addition of 11.15 ml of a 0.4 M aqueous solution of NaBH_4, _added over a period of 1 min to the vigorously stirring solution. The solution was left stirring for a further 3 h. The solvent was then removed in vacuum, the product was dried in a desiccator overnight and recovered without any further purification because a film over Celite was formed that could not be removed with CH_2_Cl_2_. The reaction scheme leading to the 1,4-benzenedimethanethiol-capped gold nanoparticles is shown in Scheme 2 (Figure 2, sample **B**; here only two nanoclusters linked by one bi-functional thiol molecule are shown).

### Instrumentation

The XRD measurements on gold nanoparticle and composite films were carried out by means of a high-resolution X-ray diffractometer (HRD3000 Ital Structures) in parallel beam optical configuration (Max-Flux™ Optical System). A fine focus X-ray tube (*λ*_Cu _= 0.154056 nm) was employed as X-ray source (X-ray generator setting of 30 mA/40 kV). For the structural characterization of the nanoparticles, a few drops of the diluted solutions are deposited on polished (100)-Si surfaces and dried in air. The X-ray diffraction patterns were recorded in glancing incidence conditions, i.e., the angle between the incident X-ray beam and the "nanoparticle film" surface was kept constant at *ω *= 1.0°, while the X-ray intensity was measured by 2*θ *of the detector-system. The average crystallite size was estimated from the diffraction peak broadening, pseudo-Voigt function fitting and applying the Scherrer's formula.

High-resolution XRD measurements were performed with a D8 Discover-Bruker diffractometer equipped with a 3 kW ceramic tube (copper anode). As primary optics a Goebel-type parabolic mirror and a two-bounce monochromator (V-grooved Ge-crystal) were used. The intensity of the scattered X-ray beams were recorded by a NaI(Tl) scintillator detector. A coupled *θ*-2*θ *movement was chosen for data collection. Concentrated nanocrystal solutions were spread on top of a silicon substrate and then the sample was allowed to dry prior the measurements. The size, shape, and crystalline structure, i.e., also *multiple twin nanoclusters *of non-crystallographic structures, of these functionalized nanoparticles were determined quantitatively by a full-pattern X-ray powder diffraction.

The morphology, shape, and size of the gold nanoparticles were also investigated by HREM and diffraction contrast imaging. All images were recorded with an FEI TECNAI G2 F30 Supertwin field-emission gun scanning transmission electron microscope (FEG STEM) operating at 300 kV and with a point-to-point resolution of 0.205 nm. TEM specimens were prepared by depositing few drops of the diluted solutions on carbon-coated TEM grids to be directly observed in the instrument.

XPS spectra were obtained by using a custom designed spectrometer. A non-monochromatized MgKαX-ray source (1253.6 eV) was used and the pressure in the instrument was maintained at 1 × 10^-9 ^Torr throughout the analysis. The experimental apparatus consists of an analysis chamber and a preparation chamber separated by a gate valve. An electrostatic hemispherical analyzer (radius 150 mm) operating in the fixed analyzer transmission (FAT) mode and a 16-channel detector were used. The film samples have been prepared by dissolving gold nanoparticles in CHCl_3 _and spinning the solutions onto polished stainless steel substrates. The samples showed good stability during the XPS analysis, preserving the same spectral features and chemical composition. The experimental energy resolution was 1 eV on the Au 4f_7/2 _component. The resolving power Δ*E*/*E *was 0.01. Binding energies (BEs) were corrected by adjusting the position of the C1s peak to 285.0 eV in those samples containing mainly aliphatic carbons and to 284.7 eV in those containing more aromatic carbon atoms, in agreement with the literature data [22]. The C1s, S2p_3/2_, and Au4f_7/2 _spectra were deconvoluted into their individual peaks using the *Peak Fit *curve-fitting program for PC. Quantitative evaluation of the atomic ratios was obtained by analysis of the XPS signal intensity, employing Scofield's atomic cross section-values [23] and experimentally determined sensitivity factors.

## Results and discussion

### Benzylthiol-capped gold nanoparticles

The modified two-phase synthesis procedure, reported in Scheme 1 (Figure 1), was applied to produce benzylthiol-protected gold nanoparticles. As a comparison, the same thiol was used in a one-phase reaction scheme (synthesis ii), in analogy to what reported in the literature for similar compounds [21]. In general a better performance of the reaction, in term of quality of nanoparticles products, was observed with the two-phase reaction (synthesis i) and different Au/S molar ratios were tested in order to achieve a fine tuning of the nanoparticles sizes. As it can be observed, when the Au/S molar ratio increases, the mean diameter of the nanoparticles increases.

**Figure 3:**
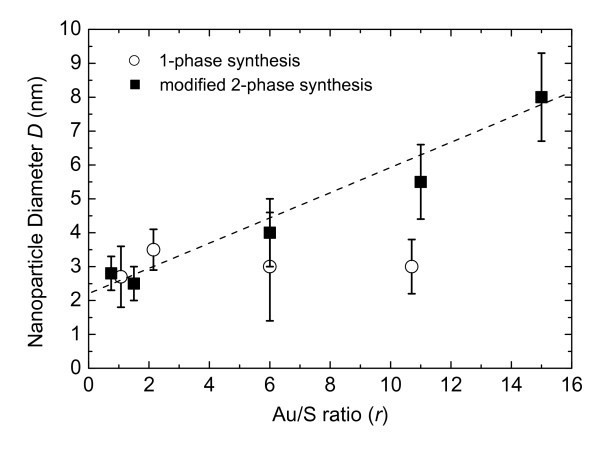
**The benzylthiol-capped gold nanoparticle diameter versus the Au/S molar ratio (*r*) as prepared by the one-phase synthesis (open circles) and the modified two-phase synthesis (filled squares)**.

In order to check the efficiency of the synthetic method, the one-phase synthesis was also carried out on the same thiol, by varying the Au/S molar ratio. Unexpectedly, the AuNP size does seem to be affected by the Au/S molar ratio. The results for the two synthesis approaches are summarized in Figure 3.

**Figure 4:**
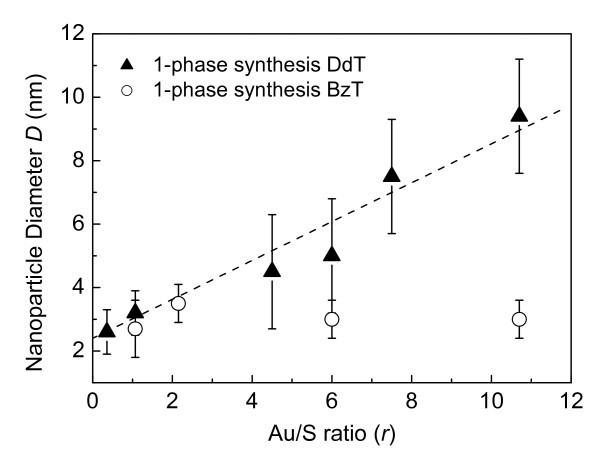
**The average diameter of the dodecanethiol (DdT, filled triangles) and benzylthiol (BzT, open circles) functionalized gold nanoparticles versus the Au/S molar ratio (*r*) by using the one-phase synthesis**.

In order to achieve a better understanding of the role of the surfactant molecule in the gold colloidal synthesis, our attention was also focused on the results obtained for dodecanethiol-capped gold nanoparticles, previously studied by our research group [21]. In that case, a modulation of the AuNP size was easily achieved by increasing the Au/S molar ratio either with one-phase or with two-phase synthesis. A linear increase of the particle sizes was observed for the pivot dodecanethiol-capped gold nanoparticles by increasing the Au/S molar ratio (Figure 4).

From this comparative study we can argue that the one-phase synthesis yields dodecanethiol-capped gold nanoparticles with tuned size, while no appreciable size modulation is achieved by using benzylthiol as surfactant. However, in the case of benzylthiol, by choosing properly the Au/S molar ratio, the modified two-phase synthesis allows to tune the diameter of the gold nanoparticles between 2 and 8 nm. Recently, efforts have been made to develop one-phase syntheses in which the reduction of metal takes place homogeneously in a selected organic solvent rather than at the two-phase interface [24]. Different reducing agents have been tested, such as amine-borane complexes and metallic nanoparticles with a narrow size distribution can be obtained, in a single step on a gram scale [25]. In our approach, Li(C_2_H_5_)_3_BH (superhydride) was used as reducing agent [26] and a lower size modulation of the BzT-stabilized nanoparticles has been observed with respect to the two-phase synthesis, although low control over the growth of nanoparticles occurred, probably due to the strong reducing effect of superhydride.

### Benzylthiol-stabilized nanoparticles characterization

**Figure 5:**
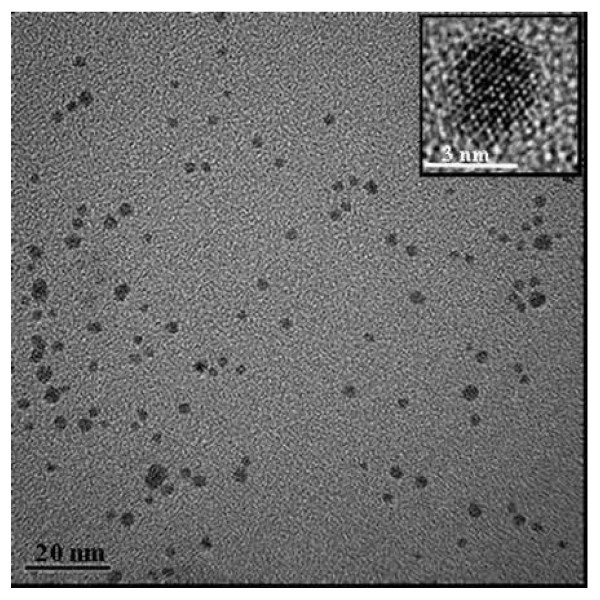
**Bright field TEM images of benzylthiol-gold nanoparticles prepared with Au/S molar ratio of 0.75:1 (sample **A1**)**. The inset shows a high-resolution image of a single Au nanoparticle of cuboctahedral structure (Ø = 3 nm); the (200) lattice fringes are well observed.

In order to deeply characterize BzT-stabilized Au nanoparticles, TEM, XRD, and XPS analyses were carried out. Figure 5 shows the TEM micrograph of the sample **A1**, representative of the samples obtained with two phases synthesis, prepared with a Au:S molar ratio 0.75:1; benzylthiol-capped gold nanoparticles are well separated and no aggregation is observed. The nanoclusters exhibit a spherical shape, and the size distribution is narrow showing a mean size of 2.8 ± 0.5 nm. The inset of Figure 3 shows a high-resolution image of a single Au nanocrystal (∅ = 3 nm) of spherical shape. Here, the (200) lattice fringes are well observed demonstrating the crystalline structure of the particle. In addition, no twinning of the crystallite is observed indicating that the nanocrystal is a single crystal (monocrystal).

High-resolution X-ray powder diffraction patterns were recorded and an accurate theoretical analysis was performed by using a quantitative whole-profile-fitting least squares data analysis technique that considers monatomic face-centered cubic (f.c.c.)-derived non-crystallographic nanoclusters of spherical shape. This advanced characterization method reveals the coexistence of cuboctahedra, formed by Au fcc crystals, Au icosahedra and decahedra (also known as *multiple twin particles*, MPTs) characterized by five-fold axes of symmetry, and allows to determine also the average particle size, size distribution and size-dependent strain of the gold nanoparticles [27]. For example, the results on the mass fraction and size distribution of the cuboctahedral, decahedral, and icosahedral crystalline structures of the benzylthiol-capped gold nanoclusters of sample **A1 **are summarized in Table 1. The average size for all the three structures (cuboctahedral, decahedral, and icosahedral) is about ∅ = (2 ÷ 2.5) nm and the size distribution is very narrow. In addition, the mass fraction for the three different structures is very similar. The XRD results are in excellent agreement with the TEM observations reported in Table 1.

**Table 1:** Mass fractions and particle sizes for the gold cuboctahedral, decahedral, and icosahedral structures of sample A1 as determined from the quantitative XRD analysis

Sample A1	Cuboctahedra	Decahedra	Icosahedra
Mass fraction (%)	36.9310	29.3696	33.6992

Particle size (nm)	2.2 ± 0.3	2.0 ± 0.1	2.0 ± 0.3

In general we observed that, by using the two-phase synthesis, a high mass fraction of the cuboctahedral crystalline structure can be obtained (the mass fractions of three structure types are about the same) and in addition, a better modulation of stabilized gold nanoparticle sizes in the range (2.5 ÷ 9) nm, when Au/S molar ratios have been varied, is achieved.

At higher Au/S molar ratios the nanoparticles are less separated and particle aggregation phenomena occur. The importance of Au/S ratios, that highly monodispersed nanoparticles are obtained upon addition of excess of thiol ligand, has been extensively highlighted in a recent study [15].

**Figure 6:**
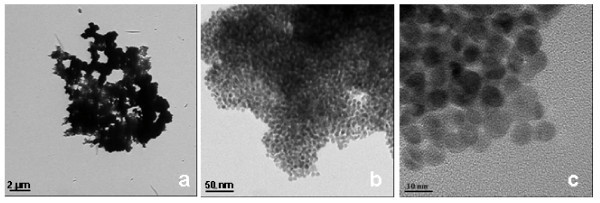
**Bright field TEM images of benzylthiol-gold nanoparticles prepared with Au/S molar ratio 15:1 (**A4**) taken with different magnification**. The particle agglomeration is well pronounced (a) and only very few isolated particles can be observed (b, c). The average particle size is about 9 nm (c).

The aggregation phenomena become evident for increasing Au/S molar ratio values as shown in Figure 6; in fact a few number of BzT molecules constitute the stabilizing shell around the metal nanoparticles and aggregation occurs. The TEM images (a), (b), and (c) are taken at increasing magnification of the sample **A4 **prepared with Au/S molar ratio 15:1. The TEM analysis clearly shows that with increasing Au:S molar ratio also the particle aggregation and the mean particle size increases (diameter ≈ 9 nm) as measured on isolated nanoclusters in Figure 4c.

The chemical and electronic structure of benzylthiol functionalized gold nanoparticles was further analyzed by XPS. The electronic structure of the nanostructured assembly is affected by nanoparticles size and their dispersion. XPS spectra of Au4f_7/2 _and S2p_3/2 _core level signals have been collected on samples obtained choosing different Au/S molar ratios in the synthetic procedure. In the following we report the results for sample **A1**, which are representative for all the samples **A **with exception of the peak intensity ratio that is related to the amount of surfactant molecules attached to the gold nanoparticle and depends on the Au/S ratio used for the synthesis. The core level binding energy (BE) and full-width at half-maxima (FWHM) are reported for C1s, Au4f and S2p core levels are summarized in Table 2.

**Table 2:** Experimental XPS data for sample A1 are summarized

Core levels	BE (eV)	FWHM (eV)	Assignation
C1s	284.70	1.80	Aromatic C
	286.62	1.80	C-S
S2p_3/2_	161.84	1.93	S-Au
Au4f_7/2_	83.69	1.53	Au(0)
	85.04	1.53	Au-S

**Figure 7:**
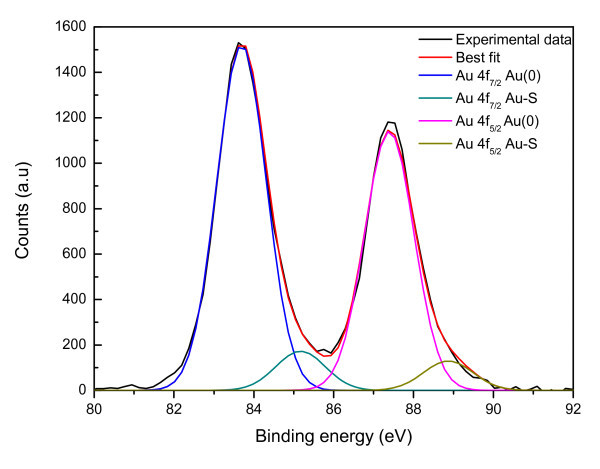
**XPS spectrum close to the Au4f peaks of sample A1**.

The C1s and S2p BE values are quite close for all the analyzed samples, showing that the size of gold nanoparticles does not affect the linked thiol chemical structure. Considering for example sample **A1**, two contributions were found by curve-fitting analysis of the two spin-orbit components of the Au4f signal (Figure 7). The Au4f_7/2 _component of the peak at lower BE values (83.69 eV) is attributed to metallic gold, the latter Au4f_7/2 _component at BE = 85.04 eV can be associated to the Au atoms that are covalently bonded to sulfur terminal groups of benzylthiol. As shown in Table 2, S2p BE value is of about 161.8 eV, fully consistent with sulfur atoms chemically interacting with metals [28]; furthermore, the semiquantitative analysis indicates that Au_Au-S_/S ratio is of about 0.75, thus indicating that nearly all thiol terminal groups are grafting the gold nanoparticle. The same characterization has been carried out for **A2-4 **samples and a fairly good agreement in semiquantitative XPS analysis has been observed.

These results clearly indicate that sulfur atoms of the benzylthiol molecules are covalently bonded to the gold atoms on the nanoparticle surface.

### 1,4-Benzenedimethanethiol-capped gold nanoparticles

In the synthesis of gold nanoparticles the bi-functional thiol 1,4-benzenedimethanethiol has been used, as reported in (Scheme 2, Figure 2). The use of a bi-functional thiol, having a sulfur atom at both ends of the ligand, would in principle allow having a double linkage with two gold nanoparticles. Consequently, the aim of using a bi-functional thiol is the possibility of obtaining a 2D or 3D network of functionalized gold nanoparticles.

In this way, the passivation molecule plays the role of a bridge rather than a simple capping agent, allowing to link more nanoparticles together forming a complex network. Considering our best result in the stabilized BzT-AuNPs, a pivot reaction has been tested with BDMT using Au/S molar ratio 0.75/1, likewise.

#### 1,4-Benzenedimethanethiol-stabilized nanoparticles characterization

**Figure 8:**
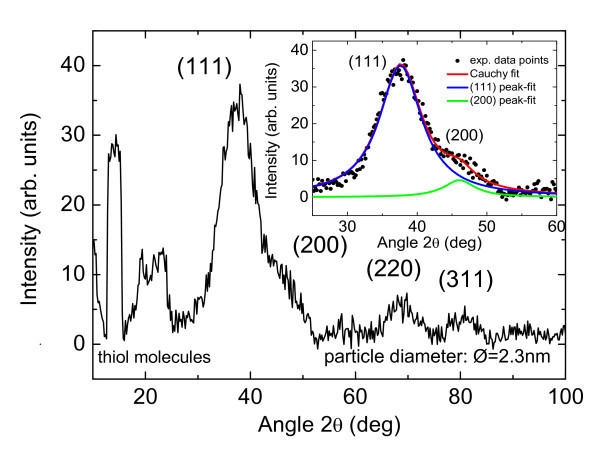
**Experimental X-ray diffraction pattern of sample B**. The average Au particle size is 2.3 nm. The inset shows the experimental (111) and (200) Au peaks together with the fitted curves.

The X-ray diffraction pattern of a sample with Au/S ratio of 0.75:1 exhibits the characteristic (111), (200), (220), and (311) Bragg peaks of the gold nanocrystals (Figure 8). The inset shows the experimental (111) and (200) Bragg peaks and the fitted curves (Cauchy functions). By using Scherrer's formula, from the full-width-of-half-maxima (FWHM) of the fitted curves, an average particle diameter of about ∅ = 2.3 nm is obtained. The diffraction pattern shows also Bragg-like peaks at small scattering angle (2*θ *<30°). These peaks are probably due to the non-reacted bi-functional thiols that are trapped within the network and cannot be easily removed by washing or by other purification methods without damaging or destroying the network itself.

**Figure 9:**
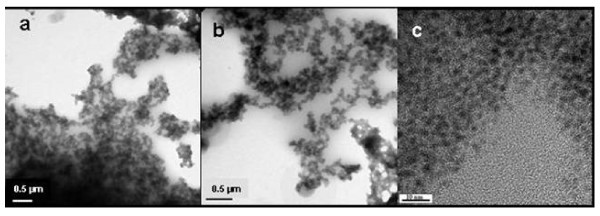
**Bright field TEM images of sample B**. The images **(a) **and **(b) **are taken at low magnification and from two different regions of the sample in order to get an idea on how nanoparticles aggregate and form chains and develop a 3D network. The image **(c) **is taken in high resolution mode and evidences the spherical shape of the particles with average and uniform size of Ø = 3 nm and their dense packing.

TEM investigations were performed on samples obtained by casting a drop of the solution on a grid. Since we can expect that a three-dimensional network of gold nanoparticles is formed, it is very important to put a "thin" film, transparent to the electron beam, on the grid and special care was dedicated to this topic. The TEM micrographs, images obtained at different magnifications (Figure 9a,b), show indeed a well pronounced three-dimensional network. At high magnification, individual nanoclusters can be observed and the distance between the gold nanoparticles seems to be very regular (Figure 9c), as expected by the bi-functional linkage. It can be estimated that the average size of the nanoparticles corresponds to a value of about 3 nm (considering that the nanoparticles are almost spherical). These data are in good agreement with the XRD measurements. The high-resolution image suggests also that the particle distribution size is quite narrow (Figure 9c). These results show that a three-dimensional network of gold nanoclusters has been obtained in the case of the bi-functional thiol molecule, in analogy to literature reports [29]: size-uniform spherical assemblies of 5-8 nm gold colloids in toluene have been obtained by cross linking the colloidal particles using alkanedithiols.

The formation of a network was already observed during and immediately after the synthesis. The gold nanoparticles aggregated in a dark flocky structure (typical dimension of approximately 1 ÷ 2 cm^3^) that remained suspended in the solution for long time. In fact, after several hours this flocky structure was deposited on the bottom of the glassware by flowing slowly the solution. After drying, this "macroscopic" structure "collapsed". However, as demonstrated by TEM observations, at the microscopic scale, the network was still preserved.

The chemical and electronic structure of 1,4-benzenedimethanethiol-functionalized gold nanoparticles was studied by XPS and the Au4f_7/2 _and S2p_3/2 _core level signals have been collected. In Table 3, the core level BE and FWHM are summarized for the C1s, Au4f, and S2p core levels of the sample **B**.

**Table 3:** Experimental XPS data for sample **B **are summarized

Sample B	BE (eV)	FWHM (eV)	Assignation
C1s	285.00	1.67	Aromatic C
S2p_3/2_	161.81	1.56	S-Au
Au4f_7/2_	83.53	1.66	Au(0)
	85.35	1.66	Au-S

**Figure 10:**
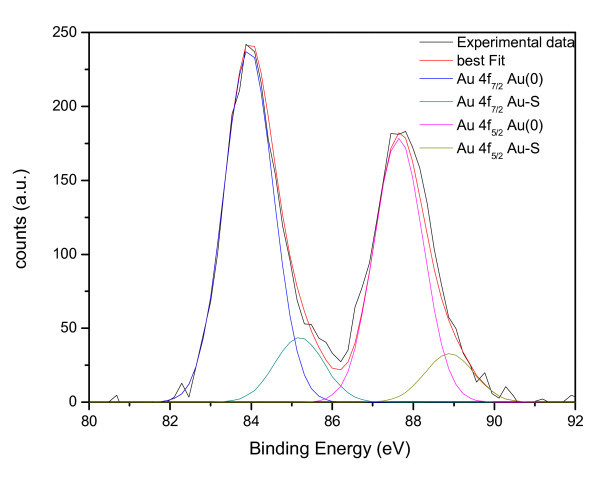
**XPS spectrum shows the Au4f peaks of sample B**.

The curve-fitting analysis of the two spin-orbit components of the Au4f signal is shown in Figure 10. Two contributions were found, similarly to the spectra of the compounds benzylthiol-capped gold nanoparticles, above discussed. Also in this case, Au4f signal is structured and two pairs of spin-orbit components are founded; the first Au4f_7/2 _component at BE = 83.53 eV is associated with metallic gold atoms, while the second Au4f_7/2 _component at higher BE values (85.35 eV) is assigned to the Au atoms that are covalently bonded to sulfur terminal groups of benzylthiol. As shown in Table 3 S2p_3/2 _signal arises at about 161.81 eV, a BE value that is fully consistent with those reported in the literature for sulfur atoms bonded to metals, as already discussed for sample **A1**. The Au_Au-S_/S ratio value for this sample, evaluated by means of XPS semiquantitative analysis, is of about 0.90. This suggests that, as for sample **A1**, nearly all thiolate terminal groups are grafting the gold nanoparticle.

The results about the structure, size, and shape obtained by TEM and XRD measurements show the presence of a network of functionalized gold nanoparticles, which distribution had an appreciable order. Preliminary micro-analytical (XPS) investigations support the formation of the covalent bond between gold and sulfur. These results open the way to further studies for the achievement of "interconnected" structures (network of thiol-linked gold nanoparticles).

## Conclusions

Benzylthiol-functionalized gold nanoparticles were synthesized by a modified two-phase method, in which a purification procedure improved the structural and morphological quality of the gold nanoparticles. A tuning of the particle size in the range 2.5-9 nm has been achieved by varying Au/S molar ratio, and high resolution XRD patterns were analyzed with theoretical models, and the coexistence of cuboctahedra, icosahedra, and decahedra gold nanoparticles, i.e., *multiple twin particles*, has been assessed. A one-phase synthesis in THF was also carried out.

The 1,4-benzendimethanethiol surfactant was used with the aim to obtain a linkage between close gold nanoparticles in order to form networks. The experimental results indicate that a very compact network structure of densely packed gold nanoparticles was achieved. XPS studies allowed studying the electronic structure of both systems, revealing the chemical interaction between sulfur atoms and gold atoms on the surface. These studies open a new way for the synthesis and fabrication of a 3D gold nanoparticle network structure for optoelectronic applications.

## Abbreviations

BDMT: 1,4-benzenedimethanethiol; BEs: binding energies; BzT: benzylthiol; FAT: fixed analyzer transmission; FWHM: full-width at half-maxima; HREM: high-resolution transmission electron microscopy; MPCs: monolayer-protected gold-nanoparticles thiolates; RT: room temperature; TOAB: tetraoctylammonium bromide; XPS: X-ray photoelectron spectroscopy; XRPD: X-ray powder diffraction XRD.

## Competing interests

The authors declare that they have no competing interests.

## Authors' contributions

FV: conceived of the study, carried out the experimental analyses and drafted the manuscript, IF: conceived the study, and participated in its design and coordination, CB: performed the XPS analyses, EP: performed the HRTEM characterizations, LT: performed the XRD analyses, conceived the study, and participated in its design and coordination, MVR: conceived the study, and participated in its design and coordination, GP: participated in the design of the study and performed the XPS analyses, CG: performed the HRXRPD analyses.
